# Supplementary material for: The causal role of circulating inflammatory markers in osteoporosis: a bidirectional Mendelian randomized study
Source: Front Immunol. 2024 Jul 18;15:1412298. doi: 10.3389/fimmu.2024.1412298 (PMC11291241; doi:10.3389/fimmu.2024.1412298)
Supplement: Supplementary file 1 [file DataSheet_1.zip › supplementary material/CIM-OSTEOPOROSIS /GCST90274828.csv_forest.pdf]

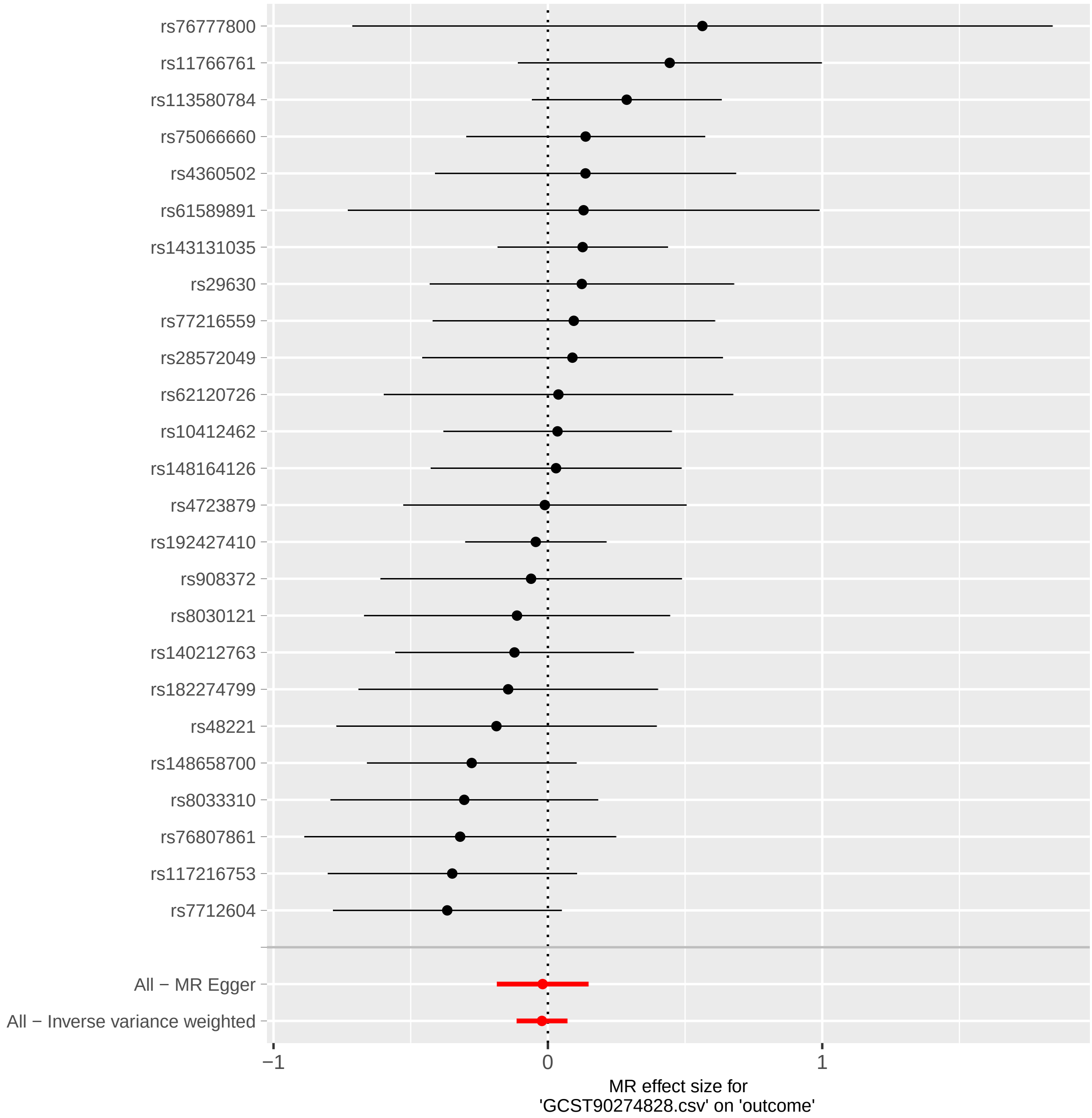
rs76777800
rs11766761
rs113580784
rs75066660
rs4360502
rs61589891
rs143131035
rs29630
rs77216559
rs28572049
rs62120726
rs10412462
rs148164126
rs4723879
rs192427410
rs908372
rs8030121
rs140212763
rs182274799
rs48221
rs148658700
rs8033310
rs76807861
rs117216753
rs7712604
All − MR Egger
All − Inverse variance weighted
−1
0
1
MR effect size for
'GCST90274828.csv' on 'outcome'
